# DNA Typing of *Mycobacterium bovis* Isolates from Badgers (*Meles meles*) Culled from Areas in Ireland with Different Levels of Tuberculosis Prevalence

**DOI:** 10.1155/2012/742478

**Published:** 2012-04-22

**Authors:** Claire Furphy, Eamon Costello, Denise Murphy, Leigh A. L. Corner, Eamonn Gormley

**Affiliations:** ^1^Central Veterinary Laboratory, Department of Agriculture, Food, and the Marine, Backweston Campus, Celbridge, Co. Kildare, Ireland; ^2^School of Veterinary Medicine, University College Dublin (UCD), Dublin 4, Ireland

## Abstract

Badgers (*Meles meles*) have been implicated in the transmission of *Mycobacterium bovis* infection to cattle in Ireland and UK. Recent studies in Ireland have shown that although the disease is endemic in badgers, the prevalence of disease is not uniform throughout the country and can vary among subpopulations. The extent to which the prevalence levels in badgers impact on the prevalence in cattle is not known. Previously, DNA fingerprinting has shown that *M. bovis* strain types are shared between badgers and cattle, and that there are a large number of strain types circulating in the two species. In this study we have carried out spoligotyping and variable number tandem repeat (VNTR) analysis of *M. bovis* isolates from two groups of badgers, representing a wide geographic area, with different tuberculosis prevalence levels. The results of the typing show that there is no geographic clustering of strain types associated with prevalence. However, two VNTR profiles were identified that appear to be associated with high- and low-prevalence *M. bovis* infection levels, respectively. In addition, spoligotyping and VNTR analysis has provided evidence, for the first time, of multiple infections of individual badgers with different *M. bovis* strains.

## 1. Introduction

In the Republic of Ireland (RoI) and the UK tuberculosis (TB) is present in badger populations [1]; infected badgers are considered as a maintenance host and are directly implicated in the transmission of *Mycobacterium bovis* to cattle [2]. In RoI, as part of a medium-term strategy for the control of TB in cattle, badgers are removed (focal culling) when an epidemiological investigation associates a cattle herd TB breakdown with the presence of infected badgers. Studies using comprehensive postmortem examination and bacterial culture of tissues have found an infection prevalence of 36–50% in these culled badgers [3] (Corner, unpublished).

In a recent comparative study we determined the infection prevalence in badgers in areas with historically and consistently low prevalence of infection in cattle [4]. Badgers were removed from geographically dispersed sites and all were examined using detailed postmortem and bacteriological procedures. A significantly lower prevalence of *M. bovis* infection was found in these badgers than in badgers removed during focal culling. While the results validated the use of cattle as sentinels for TB in badgers, they also raised questions on the nature of *M. bovis* infection in badgers in high prevalence and low prevalence populations. One possibility for the different prevalence rates is that the strains of *M. bovis* circulating in these populations differ in virulence and in their potential for transmission from badger to badger, and badger to cattle. Although very little is known about the virulence potential of field isolates of *M. bovis*, DNA fingerprinting of strains has been used to study the dynamics of TB in animals and to investigate links between infections in farmed and wild species [5, 6]. These studies have revealed many different strains circulating in cattle populations in RoI and the UK although detailed analysis has revealed that over 99% of strains originate from a single clonal complex named Eu1 [7]. In previous studies, the genotyping of 452 isolates of *M. bovis *by restriction fragment length polymorphism (RFLP) analysis in RoI revealed that the most prevalent RFLP types were widely distributed and present in both cattle and badgers [8]. The relationships between the strains isolated from cattle and badgers over large areas revealed that badgers and cattle tended to have similar strains, consistent with the sharing of *M. bovis* strains within an area and providing evidence of cross-species transmission [9].

The development of molecular strain typing techniques for the differentiation of *M. bovis* strains has greatly enhanced the ability to conduct epidemiological investigations of disease transmission in wildlife and livestock [10]. In RoI the combination of spoligotyping and MIRU-VNTR typing has proved to be superior to either test alone in revealing the diversity of *M. bovis* strains circulating in cattle and badgers [11]. In the present study we applied a combination of spoligotyping and MIRU-VNTR typing of *M. bovis* isolates from badgers in high-and low-prevalence areas of cattle TB; the objective was to investigate whether the same or dissimilar strain types were associated with the infection prevalence level in these populations.

## 2. Materials and Methods

### 2.1. Selection of Badgers from Different Prevalence Populations

*Low Prevalence Group (LP).* The detailed selection process to identify badger setts in the areas of the country with the lowest cattle TB herd prevalence has been described elsewhere [4]. Briefly, the process exploited the Irish national bovine disease database (animal health computer system, AHCSI), the Land Parcel Identification System (LPIS) geographic database, and Geographical Information Systems (GIS) analytical software. Badgers were identified in areas with herds where historically there was a low prevalence of TB in cattle (<2 standard reactors in the previous 5 years). Because the badger is a protected species in Ireland, the opportunity to cull animals is severely restricted in the absence of strong indications of the presence of disease. As LP areas do not qualify for routine badger culling, we were only granted access to LP area badgers on a very limited basis, and we were restricted to collect one badger per site with active setts.

 Areas (*n* = 198 low prevalence sites) were identified in 24 different counties. The areas were surveyed for badger setts or signs of badger activity, and 138 areas were deemed to be active. Trapping was conducted in these areas, and a single badger was removed from 101 sites in the vicinity of setts with badger activity. The badger was then euthanized and subjected to a detailed necropsy with the collection of pools of tissues for bacterial culture. Infected badgers were identified in 10 different counties giving an infection prevalence of 15.8% in these badger populations.

*High Prevalence Group (HP)*. For comparative purposes, badgers (*n* = 215) were obtained from culling operations associated with cattle herd breakdowns and were sourced from areas across 16 different counties in RoI [3]. The social group sizes ranged from 1 to 7 with a mean of 1.62 and a median of 1. There was no significant difference in infection prevalence between social groups of different sizes [3]. The *M. bovis* infection prevalence in this population was 36.3%.

### 2.2. Postmortem Examination

Captured badgers were anaesthetised with ketamine hydrochloride (0.1 mL/kg) and medetomidine (Domitor; 0.1 mL/kg) and then were euthanased with an overdose of intravenous sodium pentabarbitone. The badgers were subjected to a detailed necropsy including examination for bites or wounds. For the post mortem examination, the carcase was placed in dorsal recumbency on a downdraft table (Astec Microflow). To minimise the risk of cross-contamination, separate sterile instruments were used to expose lymph nodes, to open the abdominal and thoracic cavities, and to expose and dissect free-lymph nodes from surrounding fat and connective tissue, and to collect sections of visceral organs. From each badger, 20 separate lymph node samples and samples of kidney, spleen and liver were cultured as pools (head, carcase and thoracic lymph nodes, abdominal lymph nodes and organs, and lungs) and any bite wounds, subcutaneous abscesses or suspect gross lesions were cultured separately [3, 4]. All samples were stored at −20°C prior to culture.

### 2.3. Culture of *M. bovis*, DNA Extraction, and Spoligotyping

Samples were cultured on selective mycobacterial media as described by Murphy et al. [3, 4]. For the purpose of this study, a badger was considered infected when *M. bovis* was isolated by bacterial culture from any sample. Multiple colonies generated from each tissue sample were scraped from the solid media and transferred to a single microtube containing 500 *μ*L of phosphate-buffered saline with Tween 20 (PBS-Tw) (Sigma Aldrich, Wicklow, Ireland). DNA was extracted by heat lysing of cells as described by McLernon et al. (2010) [11]. DNA template was stored at −20°C. Spoligotyping was performed according to the method described by Kamerbeek et al. [12] except that a digoxigenin labeling and detection system (Roche Diagnostics, West Sussex) was used. Spoligotype patterns were given the names assigned in the *M. bovis *spoligotyping database at http://www.mbovis.org/.

### 2.4. VNTR Typing

VNTR typing was performed using the six loci; 2163a (alternate name, QUB 11a), 2163b (alternate name, QUB 11b), 2165 (alternate name, ETR A), 2996 (alternate name, MIRU 26), 4052 (alternate name QUB26) and 1895. The six genomic loci were amplified separately using the primers and polymerase chain reaction (PCR) procedures described by McLernon et al. [11].When the PCR was complete, the amplified products were stored at −18°C until analysed using the MegaBACE 1000 (GE Healthcare Life Sciences) as described by McLernon et al. [11].

### 2.5. Statistical Analysis

The comparisons of the strains in badgers were made between the areas, and not the individuals within the areas. The data were analysed using GraphPad Prism (GraphPad Software, USA, http://www.graphpad.com/). Chi squared test was used to analyse the geographic distribution of spoligotypes.

## 3. Results

### 3.1. Geographic Distribution of Badgers and Distribution of Infection within Badgers

In both high (HP) and low (LP) prevalence populations, infected animals were obtained in the vicinity of active setts over a wide geographic area, 16 counties for the HP group and 10 counties for the LP population. This minimized the impact of geographic clustering effects of infection and particular strain types. A total of 51 isolates were typed from 36 badgers in the HP population (average 1.4 isolates per badger, range 1–3) and 42 isolates from 16 badgers in the LP population (average 2.6 isolates per badger, range 1–5). Two or more isolates were obtained from 20 badgers: 2 tissue pools were positive in 11 badgers, 3 pools in 2 badgers, and ≥4 pools in 7 badgers. Five badgers were coinfected with two different strains, 3 from the HP group, and 2 from the LP group.

### 3.2. Spoligotyping of *M. bovis* Isolates in HP and LP Badgers

There were 9 different spoligotypes identified among the badgers, 7 types in the 36 HP badgers, and 4 among the 16 LP badgers. SB0140 was the most common spoligotype across both badger populations and apart from SB0130 the only spoligotype common to the two groups (Table 1). There was no evidence of geographic clustering of SB0140 as it was identified in all regions where the badgers for each group were sourced. There was no significant difference (*χ* ^2^ test, *P* > 0.05) in the proportion of badgers infected with this spoligotype between the groups, it being present in 13 of the 16 (81.3%) badgers of the LP population, and 24 of the 36 (66.7%) badgers of the HP group.

### 3.3. VNTR Analysis of *M. bovis* Isolates

The panel of 6 VNTR loci subdivided the 93 isolates into 22 strain types, there were 17 VNTR types in the badgers of the HP group and 9 VNTR types in the LP group. Among the group of strains bearing spoligotype SB0140, there were a total of 17 different VNTR types identified (Table 2). Thirteen of these VNTR profiles were identified in 24 badgers belonging to the HP group, while 7 were present in 13 badgers of the LP group. Two of these VNTR types were differentially represented in the HP and LP groups. VNTR type 11 3 7 5 4 4, was found in 8/13 (61.5%) badgers with SB0140 in the LP group, whereas, it was found in only 1/24 (4.1%) badgers infected with SB0140 in the HP group. VNTR type 11 4 7 5 4 4 was identified in 9/24 (37.5%) of badgers in the HP group infected with SB0140, but in only 1/13 (7.7%) of LP badgers infected with SB0140. The remaining VNTR types belonging to SB0140 and other spoligotypes were present in low numbers of badgers from each group. There was no evidence of geographical clustering of the two dominant VNTR types, both were found in badgers over a wide geographic area. 

### 3.4. Coinfection of Badgers with Multiple *M. bovis* Strain Types

Five badgers were coinfected with two different strains, 3 from the HP group and two from the LP group (Table 3). In two of the badgers the strains were differentiated by spoligotyping, while the remaining three were co-infected by strains bearing spoligotype SB0140 that were differentiated by VNTR analysis. There was no spatial relationship between any of the co-infected badgers, each animal originated from a different area. In one badger belonging to the LP group, one of the co-infecting strains was isolated from a lesion, described as an “enlarged sub-mandibular lymph node.” The distribution of infected tissues from which the co-infecting strain was isolated (Table 3) did not reveal any predilection sites.

## 4. Discussion

In RoI, prevalence studies in badgers conducted using a detailed postmortem and bacteriological examination have shown that the prevalence of infection with *M. bovis,* in areas associated with high prevalence of infection in cattle, is significantly higher than in areas with a low prevalence of infection in cattle [3, 4]. While there may be various epidemiological factors responsible for these differences in prevalence, most of which are not readily apparent, one question that can be resolved is the possibility that differences in prevalence are associated with the strains circulating in the badger population. In this study *M. bovis* strains bearing the spoligotype SB0140 were predominant among the HP and LP badger groups. In a previous study approximately 50% of 386 strains, mainly from cattle and badgers, had this spoligotype [11]. In the present study the proportion of strains bearing spoligotype SB0140 was higher especially among the LP group of badgers where 81.3% of strains were of this type. However, these differences could have been due to sampling factors and a result of the small number of isolates available for typing. Spoligotype SB0140 has a widespread distribution and is long established in RoI [11], and being found in equally high frequencies in both high and low prevalence areas, it appears not to be a defining factor in the prevalence of infection in badgers.

When VNTR analysis was used to further differentiate the strains, the highest number of VNTR profiles was found within the SB0140 strains, followed by the SB0130. This is consistent with the analysis of a much larger number of isolates from cattle, badgers, and deer [11]. The most intriguing result from the current study was the unequal representation of two VNTR profiles of the SB0140 strains between the HP and LP groups of badgers. The observation that these profiles have a wide geographic distribution suggests that their frequency of occurrence is not due to a recent local clonal expansion of a particular strain type. It is tempting to speculate that their overrepresentation in each prevalence group is indicative of virulence properties of the strains that impact on prevalence of infection in the two populations. Analysis of a much larger sample size should help clarify the national distribution of each strain type and may resolve their association with prevalence of infection in badgers. The VNTR profiles from a large sample of cattle isolates show both VNTR types were present in cattle, so the profiles are not unique to infected badgers (unpublished results). In Spain, similar strain typing studies carried out in wild animals (deer, wild boar, Iberian lynx, and fox) and cattle showed that while many *M. bovis* spoligotypes were shared between wild animals and cattle, there were spoligotypes uniquely isolated from cattle [13].

The finding of badgers coinfected with different strains *M. bovis* has not been described previously and raises questions on the pathogenesis of infection and the source of the infections. One possibility is that the strains had evolved in the badger following infection with a single strain. However, in only two of the badgers (HP108 and HP212, Table 3) could this have been possible, as there was a difference at only one VNTR locus. The spoligotypes in HP95 could not have been derived from each other by a single genetic change, and the VNTR profiles also differed at four loci. In addition, the VNTRs for LP240 differed at two loci and there were differences in both spoligotypes and VNTRs for LP307. The evidence strongly points to coinfection as the most likely explanation for multiple strains.

Coinfection with different strain types may not be related to population prevalence as it was found in each prevalence group. A badger harbouring multiple strains is probably the result of multiple transmission events as aerosol infections are the result of single bacterial clones [14]. The observation that animals are coinfected also suggests that there is limited immunity afforded by infection with the original strain that does not prevent subsequent infection. Hence, repeat exposure by either of the common routes of infection may lead to initiation of infection with additional strains. We could only identify multiple infections through differences in strain types, and multiple infections may have been more prevalent than we have reported because strains may have been indistinguishable, and badgers may have been reinfected with the strains of the same spoligotype or VNTR profile as the original infection. In addition, as samples were initially pooled for culture, any co-infecting strain present at very low levels within a pool may not have been detected.

The source of multiple infections was probably transmission from other badgers of the same social group or from immigrant badgers. In areas where badgers have been culled in successive years, it has been observed that RFLP strain profiles of *M. bovis* at particular setts can change from year to year and are most probably a consequence of inward migration of new badgers. As the LP group was obtained from areas with historically low levels of TB in cattle, it is most likely that infection resulted from badgers to badger transmission as in RoI there have been very few reports of *M. bovis* infection in other wild animals, only in wild deer [15, 16]. Given the prevalence of infection in both prevalence groups, the most plausible explanation is that multiple infections arise from contact of badgers from different territories as a result of movement between territories. While the VNTR analysis reveals the large diversity of strains in the populations, the finding of multiple strains in individual badgers might reflect the high numbers of interactions between badgers. Coinfection of wildlife (red deer, fallow deer, and wild boar) with multiple strains of *M. bovis* has been recorded in Spain, highlighting the complexity of multihost interactions and transmission of multiple strains [17]. Infection with *M. bovis *is most frequently seen in its latent form in badgers [3], and as this form of infection will have minimal impact on a badger's behaviour, the risk of acquiring new infections is not likely to be influenced by previous infections.

In infected badgers, the high prevalence of lung infection strongly supports the lungs as the principal site of primary infection and that inhalation of infectious aerosol particles is the principal mode of transmission. However, other routes including transmission *via* infected bite wounds are known to occur [3]. The distribution of different VNTR types in each co-infected badger did not provide information to deduce the possible route of infection. The transmission and maintenance of *M. bovis* in badger populations is a complex process where many factors influence within-population prevalence and rates of transmission. It is likely the infective dose of each strain may have determined its level of dissemination within the body.

The results of this study provide further evidence of extraterritorial movement of badgers and the discrimination of strains by spoligotyping, and VNTR analysis demonstrates that the interactions between badgers can result in co-infections of individual badgers with different strains. The identification of diverse VNTR profiles also suggests that different strains may be associated with the local prevalence of infection. A field vaccination trial of badgers with the BCG vaccine is currently underway [18]. Although it has been demonstrated that the BCG is effective in protecting captive badgers against experimental *M. bovis* infection, it remains to be determined if the vaccine is equally effective against the many *M. bovis* strain types found in badgers [19].

## Figures and Tables

**Table 1:** Number of badgers infected with each *M. bovis* spoligotype. *One HP badger was coinfected with SB0130 and SB0275. **One LP badger was coinfected with SB0263 and SB0140.

Spoligotype	HP	LP
SB0130	5	2
SB0140	24	13
SB0142	1	0
SB0144	1	0
SB0145	1	0
SB0146	0	1
SB0263**	0	1
SB0275*	2	0
SB0978	3	0

**Table 2:** VNTR profiles of *M. bovis* strains bearing the SB0140 spoligotype. Differentially represented strains are highlighted/italicised. *VNTR types were not unique to individual badgers (see Table 3).

VNTR	HP	LP
3 4 7 5 4 4*	3	0
3 5 7 5 4 4*	2	0
9 4 7 5 3 4	1	0
9 4 7 5 4 4	1	0
10 3 4 5 4 3	1	0
10 3 5 5 4 3	1	0
10 4 5 5 4 3	0	1
10 4 7 5 4 4*	0	1
11 3 7 5 4 4*	1	8
11 4 5 5 3 4	1	0
11 4 6 5 4 4	2	0
11 4 7 5 2 4	1	1
11 4 7 5 3 4	1	0
11 4 7 5 4 4*	9	1
11 4 7 6 3 4	2	0
6 3 7 5 4 4	0	1
6 4 7 5 4 4	0	1

**Table 3:** Coinfection of badgers with *M. bovis* strains. Tissues from which *M. bovis* was isolated: H: head, T: Thorax, C: Carcase, L: Lung, Les: lesion, A: Abdomen. Co-infecting strains are highlighted/italicised. VNTR loci are listed in order 2163a, 2163b, 2165, 2996, 4052, and 1895.

Badger	Tissue	Spoligotype	VNTR
HP95	H	SB0275	10 3 7 5 4 4
HP95	T	**SB0130**	** 3 3 7 3 3 2**
HP95	C	SB0275	10 3 7 5 4 4
HP108	H	SB0140	3 4 7 5 4 4
HP108	T	**SB0140**	** 3 5 7 5 4 4**
HP108	C	SB0140	3 4 7 5 4 4
HP212	C	**SB0140**	** 3 4 7 5 4 4**
HP212	H	**SB0140**	**11 4 7 5 4 4**
LP240	H	SB0140	10 4 7 5 4 4
LP240	L	SB0140	10 4 7 5 4 4
LP240	C	SB0140	10 4 7 5 4 4
LP240	Les	**SB0140**	**11 3 7 5 4 4**
LP307	T	SB0140	6 4 7 5 4 4
LP307	H	SB0140	6 4 7 5 4 4
LP307	L	SB0140	6 4 7 5 4 4
LP307	C	SB0140	6 4 7 5 4 4
LP307	A	**SB0263**	**10 4 7 5 4 4**

## References

[1] Dolan LA (1993). *Badgers and Bovine Tuberculosis in Ireland: A Review*.

[2] Griffin JM, Williams DH, Kelly GE (2005). The impact of badger removal on the control of tuberculosis in cattle herds in Ireland. *Preventive Veterinary Medicine*.

[3] Murphy D, Gormley E, Costello E, O’Meara D, Corner LAL (2010). The prevalence and distribution of *Mycobacterium bovis* infection in European badgers (*Meles meles*) as determined by enhanced post mortem examination and bacteriological culture. *Research in Veterinary Science*.

[4] Murphy D, Gormley E, Collins DM (2011). Tuberculosis in cattle herds are sentinels for *Mycobacterium bovis* infection in European badgers (*Meles meles*): the Irish greenfield study. *Veterinary Microbiology*.

[5] Skuce RA, Neill SD (2001). Molecular epidemiology of *Mycobacterium bovis*: exploiting molecular data. *Tuberculosis*.

[6] Haddad N, Masselot M, Durand B (2004). Molecular differentiation of *Mycobacterium bovis* isolates. Review of main techniques and applications. *Research in Veterinary Science*.

[7] Smith NH, Berg S, Dale J (2011). European 1: a globally important clonal complex of *Mycobacterium bovis*. *Infection, Genetics and Evolution*.

[8] Costello E, O’Grady D, Flynn O (1999). Study of restriction fragment length polymorphism analysis and spoligotyping for epidemiological investigation of *Mycobacterium bovis* infection. *Journal of Clinical Microbiology*.

[9] Olea-Popelka FJ, Flynn O, Costello E (2005). Spatial relationship between *Mycobacterium bovis* strains in cattle and badgers in four areas in Ireland. *Preventive Veterinary Medicine*.

[10] Durr PA, Hewinson RG, Clifton-Hadley RS (2000). Molecular epidemiology of bovine tuberculosis. I. *Mycobacterium bovis* genotyping.. *OIE Revue Scientifique et Technique*.

[11] McLernon J, Costello E, Flynn O, Madigan G, Ryan F (2010). Evaluation of mycobacterial interspersed repetitive-unit-variable-number tandem-repeat analysis and spoligotyping for genotyping of *Mycobacterium bovis* isolates and a comparison with restriction fragment length polymorphism typing. *Journal of Clinical Microbiology*.

[12] Kamerbeek J, Schouls L, Kolk A (1997). Simultaneous detection and strain differentiation of *Mycobacterium tuberculosis* for diagnosis and epidemiology. *Journal of Clinical Microbiology*.

[13] Romero B, Aranaz A, Sandoval J (2008). Persistence and molecular evolution of *Mycobacterium bovis* population from cattle and wildlife in Donana National Park revealed by genotype variation. *Veterinary Microbiology*.

[14] Richardson M, Carroll NM, Engelke E (2002). Multiple *Mycobacterium tuberculosis* strains in early cultures from patients in a high-incidence community setting. *Journal of Clinical Microbiology*.

[15] Quigley FC, Costello E, Flynn O (1997). Isolation of mycobacteria from lymph node lesions in deer. *Veterinary Record*.

[16] Dodd K (1984). Tuberculosis in free-living deer. *Veterinary Record*.

[17] Gortazar C, Torres MJ, Acevedo P (2011). Fine-tuning the space, time, and host distribution of mycobacteria in wildlife. *BMC Microbiology*.

[18] Aznar I, McGrath G, Murphy D (2011). Trial design to estimate the effect of vaccination on tuberculosis incidence in badgers. *Veterinary Microbiology*.

[19] Gormley E, Corner LAL (2011). Control of tuberculosis in badgers by vaccination: where next?. *Veterinary Journal*.

